# Intracystic papillary carcinoma of the male breast: a case report

**DOI:** 10.1186/s12957-018-1318-5

**Published:** 2018-01-23

**Authors:** Haruhito Kinoshita, Shinichiro Kashiwagi, Hitoshi Teraoka, Takuya Mori, Kenji Kuroda, Mikio Nanbara, Eiji Noda, Takaaki Chikugo, Kosei Hirakawa, Masaichi Ohira

**Affiliations:** 1 Department of Surgery, Baba Memorial Hospital, Higashi 4-244 Hamadera Funao-cho, Nishi-ku, Sakai, Osaka 592-8555 Japan; 2 Department of Surgical Oncology, Osaka City University Graduate School of Medicine, 1-4-3 Asahi-machi, Abeno-ku, Osaka 545-8585 Japan; 3 Department of Pathology, Kinki University Faculty of Medicine, Osaka-sayama, Osaka 589-8511 Japan

**Keywords:** Intracystic papillary carcinoma, Breast cancer, Male, Ultrasonography, Vacuum-assisted biopsy

## Abstract

**Background:**

Intracystic papillary carcinoma (IPC) is defined as cancer that develops from the wall of a cyst in the breast. As breast cancer in men accounts for only 1% of all breast cancers, male IPC is an extremely rare form of the disease. The present case report examines IPC in a man, along with an in-depth literature discussion.

**Case presentation:**

A 64-year-old Japanese man noticed a mass in the right breast and sought medical attention. An elastic and soft neoplastic 3-cm lesion was palpated in the right papilla. As a 1-cm solid tumor with a gradual rise from the cyst wall was confirmed within the cyst, vacuum-assisted biopsy (VAB) was performed on that site. Pathological examination of the biopsy revealed heterotypic cells with an enlarged oval nucleus forming dense papillary structures mainly of vascular connective tissue component. Contrast-enhanced computed tomography (CT) confirmed thickening of the wall that protruded outside the cyst. The preoperative diagnosis was right breast cancer (male IPC) TisN0M0 stage 0 luminal B-like. Total mastectomy and sentinel lymph node biopsy were performed. In the excised specimen, a 4.0-cm unilocular cyst was found, along with a 1-cm solid tumor with a gradual rise from the cyst wall. Pathological diagnosis of the resected specimen shared similar characteristics with the solid tumor in the cyst: notably, an oval nucleus with histologically clear nucleolus and fine granular chromatin, cylindrically shaped heterotypic cells, and the presence of basophilic cells in the papillary growth with a thin stem of fibrovasculature as the axis. Some invasion of tumor cells into the interstitium was confirmed. As such, the final diagnosis was right breast cancer (male IPC) T2N0M0 stage IIA luminal B-like. The expression of hormone receptor (ER and PgR) was high, and endocrine therapy was initiated postoperatively (20 mg/day tamoxifen). At the present time (3 months postoperation), there has not been any evidence of metastasis.

**Conclusions:**

We reported a rare case of an IPC in the male breast, along with a literature review.

## Background

Intracystic papillary carcinoma (IPC) was first reported by Brodie in 1846, and its incidence is approximately 0.26–2.0% of all breast cancers [1]. IPC is defined as cancer that develops from the wall of a cyst in the breast. As breast cancer in men accounts for only 1% of all breast cancers [2], male IPC is an extremely rare form of the disease [3–5]. The present case report examines IPC in a man, along with an in-depth literature discussion.

## Case presentation

**Fig. 1:**
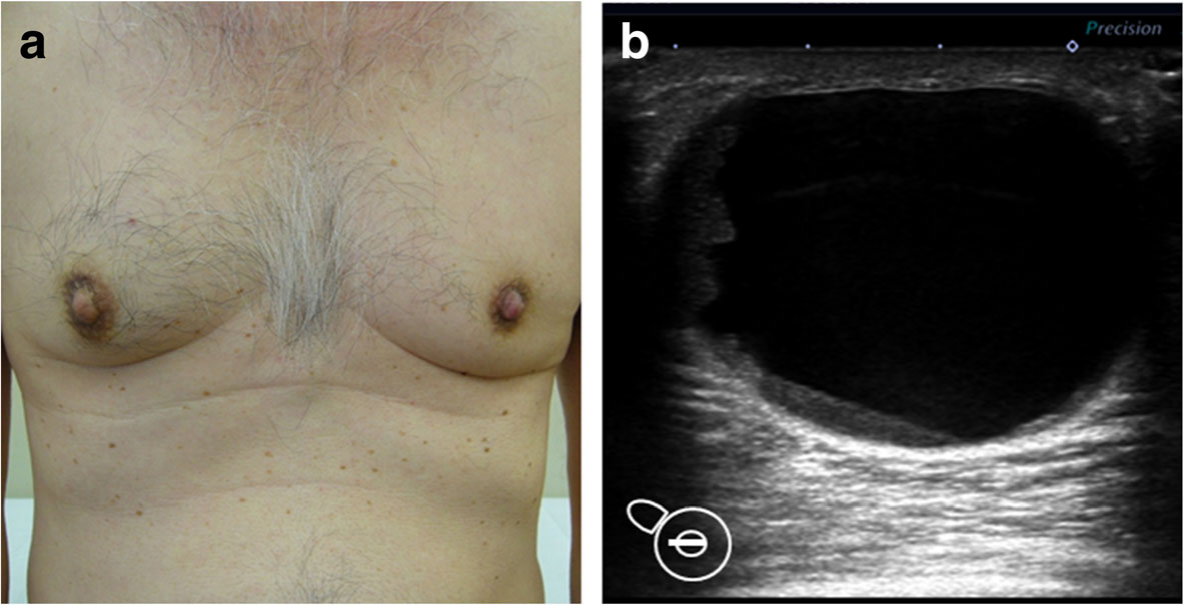
Physical and ultrasonography findings: An elastic and soft neoplastic 3-cm lesion was palpated in the right papilla (**a**). On mammary ultrasonography, a cystic lesion of 3.9 × 2.6 cm was confirmed immediately below the right papilla (mixed pattern). As a 1-cm solid tumor with a gradual rise from the cyst wall was confirmed within the cyst (**b**)

**Fig. 2:**
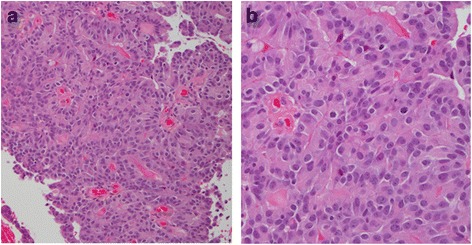
Pathological findings of vacuum-assisted biopsy: Pathological examination of the biopsy revealed heterotypic cells with an enlarged oval nucleus forming dense papillary structures mainly of vascular connective tissue components (**a** × 100) (**b** × 400)

**Fig. 3:**
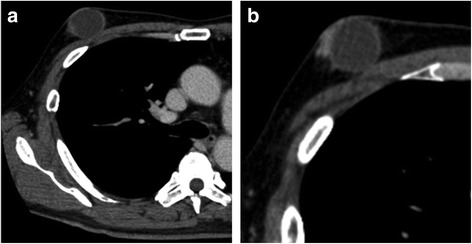
CT image findings: Contrast-enhanced computed tomography confirmed thickening of the wall that protruded outside the cyst (**a**, **b**)

**Fig. 4:**
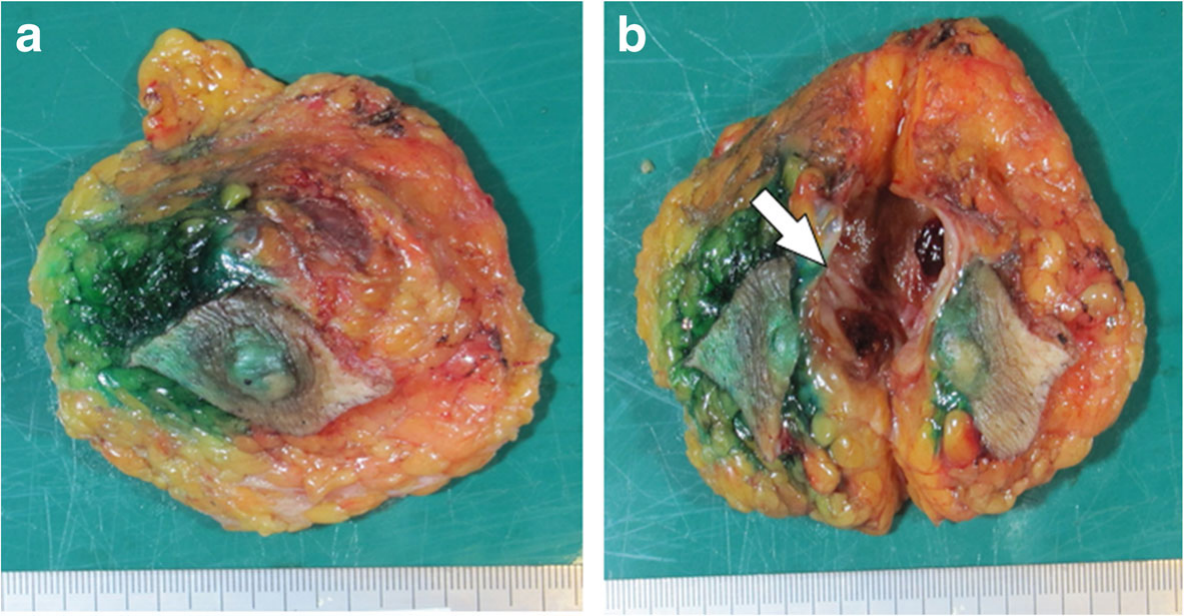
Macroscopic diagnosis of the resected specimen: In the excised specimen, a 4.0-cm unilocular cyst was found, along with a 1-cm solid tumor with a gradual rise from the cyst wall (arrow) (**a** anterior image) (**b** split face of posterior image)

**Fig. 5:**
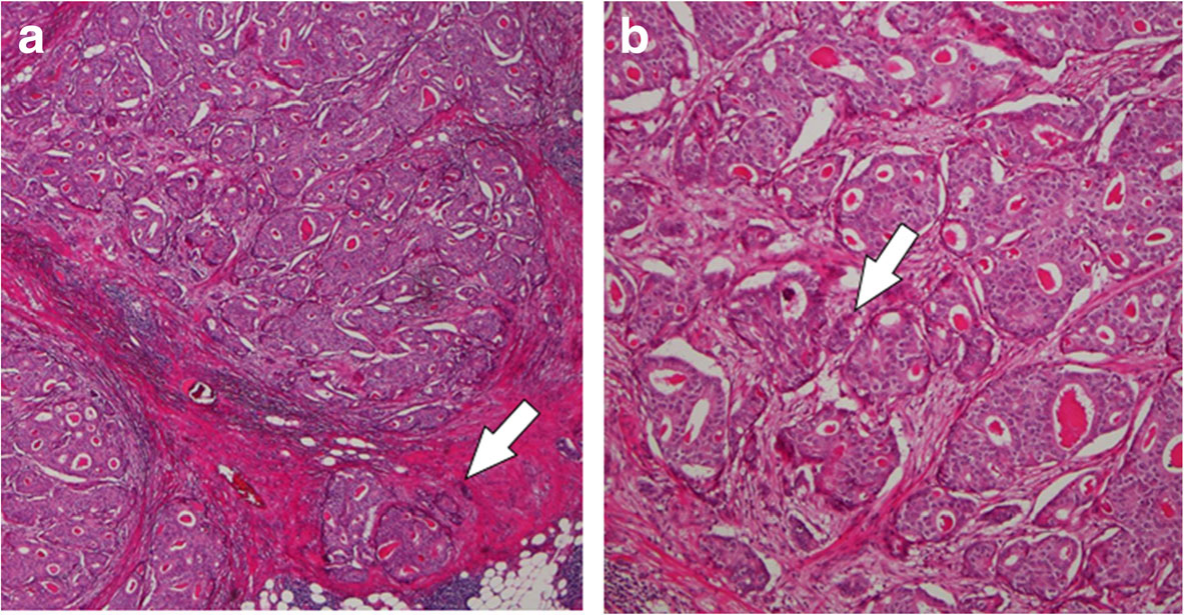
Pathological diagnosis of the resected specimen: Pathological diagnosis of the resected specimen shared similar characteristics with the solid tumor in the cyst: notably, an oval nucleus with histologically clear nucleolus and fine granular chromatin, cylindrically shaped heterotypic cells, and the presence of basophilic cells in the papillary growth with a thin stem of fibrovasculature as the axis (**a** × 100) (**b** × 400). Some invasion of tumor cells into the interstitium was confirmed (arrow)

A 64-year-old Japanese man noticed a mass in the right breast and sought medical attention. The patient’s medical records showed that he was currently undergoing treatment for alcoholic hepatitis. Physical findings confirmed gynecomastia in both breasts. An elastic and soft neoplastic 3-cm lesion was palpated in the right papilla (Fig. 1a). There were no skin lesions, and swollen lymph nodes were not palpated. Blood tests showed that tumor marker levels were normal (carcinoembryonic antigen [CEA] level of 1.6 ng/ml [normal, < 5.0 ng/ml], cancer antigen 15-3 [CA15-3] level of 7.5 IU/ml [normal, < 25.0 IU/ml], and National Cancer Center-Stomach-439 (NCC-ST-439) level of 1.0 U/ml [normal, < 4.5 U/ml]). On mammary ultrasonography, a cystic lesion of 3.9 × 2.6 cm was confirmed immediately below the right papilla (mixed pattern). As a 1-cm solid tumor with a gradual rise from the cyst wall was confirmed within the cyst, vacuum-assisted biopsy (VAB) was performed on that site (Fig. 1b). Pathological examination of the biopsy revealed heterotypic cells with an enlarged oval nucleus forming dense papillary structures mainly of vascular connective tissue components (Fig. 2a, b). There was a nucleolus in the nucleus, and some mitotic images were noted. No invasion of tumor cells into the interstitium was noted. Upon immunohistochemical staining, estrogen receptor (ER) and progesterone receptor (PgR) were diffusely positive, human epidermal growth factor receptor 2 (HER2) was negative, and the Ki 67 index was 62.5%. Contrast-enhanced computed tomography (CT) confirmed thickening of the wall that protruded outside the cyst (Fig. 3a, b). A systemic examination demonstrated that there were no metastases to the lungs, liver, or lymph nodes. Furthermore, there were no bone metastases as determined via bone scintigraphy. The preoperative diagnosis was right breast cancer (male IPC) TisN0M0 stage 0 luminal B-like. Total mastectomy and sentinel lymph node biopsy were performed. Intraoperative pathological diagnosis confirmed a lack of metastasis to the sentinel lymph node; hence, axillary lymph node dissection was omitted. In the excised specimen, a 4.0-cm unilocular cyst was found, along with a 1-cm solid tumor with a gradual rise from the cyst wall (Fig. 4a, b). Pathological diagnosis of the resected specimen shared similar characteristics with the solid tumor in the cyst: notably, an oval nucleus with histologically clear nucleolus and fine granular chromatin, cylindrically shaped heterotypic cells, and the presence of basophilic cells in the papillary growth with a thin stem of fibrovasculature as the axis. Some invasion of tumor cells into the interstitium was confirmed. In the papillary structure, p63-positive myoepithelial cells were absent and it was not biphasic (nuclear grade grade 2, nuclear atypia score 3, mitotic counts score 1) (surgical margin free) (Fig. 5a, b). ER and PgR staining were diffusely positive, HER2 staining was negative, and the Ki 67 index was 6.6%. In immunostaining, cytokeratin (CK) 20 was negative, and androgen receptor (AR) was positive. As such, the final diagnosis was right breast cancer (male IPC) T2N0M0 stage IIA luminal B-like. The expression of hormone receptor (ER and PgR) was high, and endocrine therapy was initiated postoperatively (20 mg/day tamoxifen). At the present time (3 months postoperation), there has not been any evidence of metastasis.

## Discussion

Breast cancers containing cysts can be divided into three types based on the tumor initiation site and pathological criteria: (1) necrosis at the center of noncystic breast cancers, leading to secondary cystoid degeneration (cystic breast cancer), (2) breast cancer and mammary cystic disease combined, where cancer has developed into mammary cysts and spread (combined breast cancer and cystic disease), and (3) breast cancer that has developed directly from the cystic wall; these tumors are considered IPC [6]. However, it is frequently difficult to make these distinctions, and since this particular case had a clear cystic structure and tumor was present within the cyst, it was formally diagnosed as IPC.

IPC is often discovered as an uneven mass, and without medical intervention, it tends to form relatively large tumor masses. When there is no invasion outside of the cyst wall, the malignancy is treated as Tis [7]. However, since there is a large amount of cystic components present in biopsy material, it is difficult to confirm invasion preoperatively [6]. Fine-needle aspiration cytology (FNAC) and core-needle biopsy (CNB) are usually performed, but the false negative results by cytology are relatively frequent [8]. In this case, we performed VAB to sample the rich biopsy material, but unfortunately could not confirm invasion preoperatively.

Since the prognosis of IPC after surgery is good and the incidence of local recurrence is low [9], if it is possible, partial mammary resection should be performed. However, as many cases present close to the mammary glands [4], many physicians rely on total mastectomy, similar to that performed in this case [4, 10]. With respect to the axillary operation, similar criteria to routine breast cancer surgery are used, and in this case, as there was no metastasis in the sentinel lymph node biopsy, axillary dissection was omitted.

Malignant progression in IPC is relatively low, with the majority of cases having a good prognosis, and there is no difference in prognosis between non-invasive and invasive types [4, 11]. There is currently no standard of care considering postoperative treatment for IPC since there have not been many reported cases and prognosis following routine intervention based on tumor subtype is quite good. There are no clear guidelines on IPC management. Grabowski et al. confirmed that surgery is the mainstream of treatment [12]. The prognosis of IPC is excellent with low locoregional and distant recurrence rates, so mastectomy is usually not necessary, unless it is technically inevitable [12]. Moreover, there has been no clear indication for adjuvant endocrine therapy, even among the hormone-receptor-positive patients. If there is non-invasive ductal carcinoma outside the cyst wall or micro-invasive carcinoma in the interstitium, radiation therapy and pharmacotherapy are usually implemented, but these therapies for IPC have not been extensively evaluated [13]. However, similar to normal breast cancer, treatment for the intrinsic subtype should be performed, and as most male breast cancers are hormone receptor positive, tamoxifen is often administered [14, 15]. Also, postoperative adjuvant therapy for male breast cancer is thought that it should be done according to female breast cancer [16]. Indeed, the case presented here was hormone receptor positive, and tamoxifen was administered postoperatively [17–19].

## Conclusions

In conclusion, we reported a rare case of an IPC in the male breast, along with a literature review.

## Abbreviations

AR: Androgen receptor
CA15-3: Cancer antigen 15-3
CEA: Carcinoembryonic antigen
CK: Cytokeratin
CNB: Core-needle biopsy
ER: Estrogen receptor
FNAC: Fine-needle aspiration cytology
HER2: Human epidermal growth factor receptor 2
IPC: Intracystic papillary carcinoma
NCC-ST-439: National Cancer Center-Stomach-439
PgR: Progesterone receptor
VAB: Vacuum-assisted biopsy
